# Bofutsushosan improves gut barrier function with a bloom of *Akkermansia muciniphila* and improves glucose metabolism in mice with diet-induced obesity

**DOI:** 10.1038/s41598-020-62506-w

**Published:** 2020-03-26

**Authors:** Shiho Fujisaka, Isao Usui, Allah Nawaz, Yoshiko Igarashi, Keisuke Okabe, Yukihiro Furusawa, Shiro Watanabe, Seiji Yamamoto, Masakiyo Sasahara, Yoshiyuki Watanabe, Yoshinori Nagai, Kunimasa Yagi, Takashi Nakagawa, Kazuyuki Tobe

**Affiliations:** 1 First Department of Internal Medicine, Faculty of Medicine, University of Toyama, Toyama, Japan; 2 Department of Endocrinology and Metabolism, Dokkyo Medical University, Tochigi, Japan; 3 Department of Metabolism and Nutrition, Graduate School of Medicine and Pharmaceutical Sciences for Research, University of Toyama, Toyama, Japan; 4 Department of community Medical Support, Toyama University Hospital, Toyama, Japan; 5 Department of Liberal Arts and Sciences, Faculty of Engineering, Toyama Prefectural University, Toyama, Japan; 6 Division of Nutritional Biochemistry, Institute of Natural Medicine, University of Toyama, Toyama, Japan; 7 Department of Pathology, University of Toyama, Toyama, Japan; 8 Department of Pharmaceutical Engineering, Faculty of Engineering, Toyama Prefectural University, Toyama, Japan

**Keywords:** Obesity, Obesity

## Abstract

Obesity and insulin resistance are associated with dysbiosis of the gut microbiota and impaired intestinal barrier function. Herein, we report that Bofutsushosan (BFT), a Japanese herbal medicine, Kampo, which has been clinically used for constipation in Asian countries, ameliorates glucose metabolism in mice with diet–induced obesity. A 16S rRNA sequence analysis of fecal samples showed that BFT dramatically increased the relative abundance of Verrucomicrobia, which was mainly associated with a bloom of *Akkermansia muciniphila* (AKK). BFT decreased the gut permeability as assessed by FITC-dextran gavage assay, associated with increased expression of tight-junction related protein, claudin-1, in the colon. The BFT treatment group also showed significant decreases of the plasma endotoxin level and expression of the hepatic lipopolysaccharide-binding protein. Antibiotic treatment abrogated the metabolic effects of BFT. Moreover, many of these changes could be reproduced when the cecal contents of BFT-treated donors were transferred to antibiotic-pretreated high fat diet-fed mice. These data demonstrate that BFT modifies the gut microbiota with an increase in AKK, which may contribute to improving gut barrier function and preventing metabolic endotoxemia, leading to attenuation of diet-induced inflammation and glucose intolerance. Understanding the interaction between a medicine and the gut microbiota may provide insights into new pharmacological targets to improve glucose metabolism.

## Introduction

Obesity and insulin resistance have become epidemic metabolic abnormalities, that can result in pathological complications such as diabetes, hypertension, sarcopenia and cancer^1,2^. Obesity-induced insulin resistance is associated with low-grade systemic inflammation, induced by activation of inflammatory signaling^3–7^. Thus, controlling obesity-associated inflammation has been a possible therapeutic target in the past couple of decades.

The gut microbiota is an important determinant of the metabolic and energy status. They represent a source of energy, such as short-chain fatty acids (SCFAs), that accounts for 5–10% of the total energy demand of the host^8^. Hence, germ-free mice are obesity-resistant under a high fat -diet (HFD) and start to gain weight after bacterial colonization. Transplanting the intestinal bacteria of obese individuals into germ-free (GF) mice results in obesity^9,10^. Thus, it is clear that intestinal bacteria control the host’s energy balance.

Glucose metabolism is also regulated by intestinal bacteria. For example, SCFAs regulate insulin secretion and energy metabolism and have beneficial effects on glucose metabolism^11–13^. Secondary bile acids, another set of major bacterial metabolites, are known to activate transmembrane G protein-coupled receptor 5 (TGR5) and promote secretion of glucagon-like peptide-1 (GLP-1)^14,15^. Antibiotic treatment of HFD-fed mice has been reported to improve glucose metabolism via reducing inflammatory bacterial metabolites and lipopolysaccharides^16,17^.

In addition to producing the aforementioned bacterial metabolites, intestinal bacteria also play an important role in influencing insulin resistance. They contribute to maintenance of the intestinal barrier function. When the intestinal microbiota is in a healthy condition, the tight junctions of the intestinal epithelium are dense, and a thick mucus layer is formed^18^. Recent studies have revealed that obesity and a high fat diet cause dysbiosis, characterized by decreased bacterial diversity and a decreased amount of AKK; the mucous layer of the intestinal epithelium becomes thin and the expressions of tight-junction-related proteins decrease. In this “leaky gut”, the intestinal permeability increases and the lipopolysaccharides derived from gram-negative bacteria flow into the circulation. As a result, Toll-like receptor 4 (TLR4) signaling is activated, promoting systemic inflammation^19^. In fact, in both human and rodents, obese subjects show endotoxemia, which is correlated with insulin resistance^19,20^. Therefore, it would be beneficial to identify factors that can restore the barrier function, as possible therapeutic targets for insulin resistance.

*Akkermansia muciniphila* (AKK) is a gram-negative anaerobic bacterium, accounting for 3–5% of the resident bacteria in the gut^21,22^. AKK degrades mucus, but administration of AKK increases goblet cells and thickens the mucous layer, and increases tight-junction-related protein^23^. In addition, it has been reported that administration of a purified membrane protein from AKK can improve the gut barrier function and glucose metabolism^24^. An HFD markedly decreases AKK in mice, and lower levels of AKK have been shown to be correlated with obesity and insulin resistance in humans^25^. Dietary polyphenols have been reported to promote growth of AKK and improve glucose metabolism, via increasing tight junctions in the intestinal epithelium and attenuating diet -induced inflammation^26^. Metformin widely used for patients with diabetes has also been reported to increase AKK^27^ and thicken the mucous layer of the intestine to improve glucose metabolism^28^. Recently, Plovier and colleagues reported that the cell membrane protein of AKK improves the barrier function of the intestine and glucose metabolism^24^. Furthermore, administration of AKK to obese humans has been reported to improve insulin sensitivity and several metabolic parameters^29^.

Intervention of the gut microbiota can be a novel concept in improving the pathophysiology of insulin resistance. However, at present, few Western medicines improve the underlying condition of metabolic syndrome. On the other hand, traditional herbal medicines, which are a combination of natural herbal ingredients, have been clinically used for a long time, with few side effects. Among them, Bofutsushosan (BFT) is a Japanese herbal medicine, Kampo, composed of 18 crude components (Supplementary Table 1). Clinically, BFT exerts a laxative effect since it contains sennosides derived from Rhei rhizoma and has mainly been used in Asian countries for the treatment of constipation in obese patients. It has also been reported that high doses of BFT can activate UCP-1 expression in the brown adipose tissue, decrease the plasma ghrelin level and suppress body weight gain by decreasing the visceral adipose tissue, and attenuate the progression of steatohepatitis^30–34^. However, how BFT affects the gut microbiota and contributes to metabolic improvement is not yet fully understood.

In this study, we showed that BFT modifies the gut microbiota; in particular, it increased AKK and enhanced the intestinal barrier function. As a result, HFD -induced endotoxemia and inflammation of adipose tissue was ameliorated by BFT, leading to improved insulin sensitivity and glucose tolerance. Cecal microbiota transplantation from BFT-treated mice reproduced the improvement of the glucose metabolism. These findings provide a new insight into the effects of a herbal medicine on the gut microbiota and glucose metabolism.

## Results

### BFT Improved the glucose metabolism in mice with diet-induced obesity

**Figure 1:**
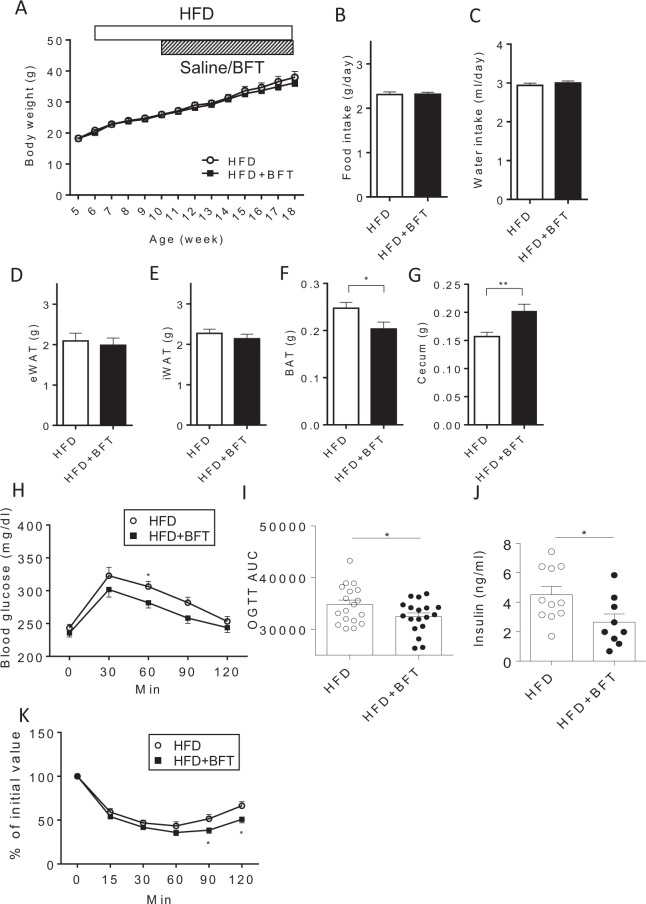
Bofutsushosan (BFT) improved glucose metabolism without affecting body weight. (**A**) Body weight of mice treated with either saline (white) or BFT (black) on an high fat-diet (HFD) (n = 8–9). Average food (**B**) and water (**C**) intake of mice between 11 and 14 weeks of age (n = 8–9). Weight of epididymal adipose tissue (eWAT) (**D**) inguinal adipose tissue (iWAT) (**E**), brown adipose tissue (BAT) (**F**) and cecum (**G**) of mice treated with either saline or BFT (18–19 weeks of age, n = 8–11). (**H**) Oral glucose tolerance test (OGTT) and (**I**) AUC of HFD-fed mice treated with saline (white) or BFT (black) for 5 weeks (n = 18). (**J**) Plasma insulin levels in 2-hour-fasted state (n = 9–11, 18 weeks of age). (**K**) Insulin tolerance test (ITT) of HFD-fed mice treated with saline (white) or BFT (black) for 8 weeks (n = 18–19). *p < 0.05, **p < 0.01 by unpaired, 2-tailed t test.

No alteration of the body weight (Fig. 1A), food and water intake (Fig. 1B,C), or weight of the epididymal adipose tissue and inguinal adipose tissue (Fig. 1D,E) were observed by BFT administration. On the other hand, BFT decreased the weight of the brown adipose tissue (BAT) (Fig. 1F), even though it exerted no effects on the expression levels of mitochondria-related genes, such as *Ucp1* and *Pgc-1α*, in the brown adipose tissue (BAT) (Supplementary Fig. 1). BFT treatment was associated with a significant increase of the cecum size (Fig. 1G). The oral glucose tolerance test (OGTT) revealed that BFT improved glucose tolerance with decreased insulin levels (Fig. 1H–J). Improvement of the insulin sensitivity was confirmed by the insulin tolerance test (ITT) (Fig. 1K). These results indicate that BFT improved glucose metabolism without affecting the body weight in mice with diet-induced obesity.

### BFT modified the Gut microbiota

**Figure 2:**
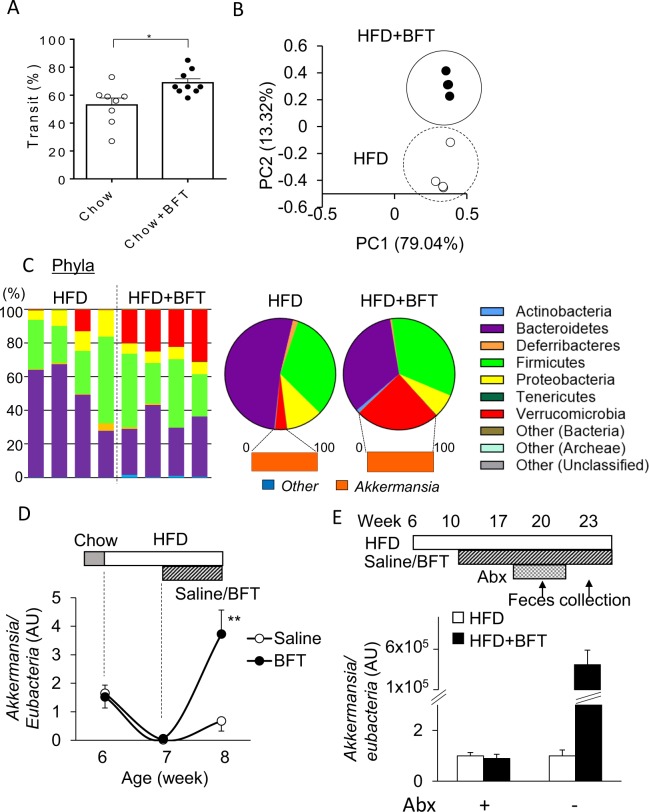
BFT modulates microbiota composition and increases *Akkermansia muciniphila*. (**A**) Migration rate of charcoal meal in the small intestine. (**B**) Principal component analysis (PCA) of fecal 16S rRNA sequencing data for mice treated with either saline or BFT on an HFD at 19 weeks of age (n = 4). (**C**) Representation of individual (left) and average (right) bacterial phyla in the fecal microbiota at 19 weeks of age (n = 4). (**D**) Fecal *Akkermansia* DNA levels normalized to eubacteria levels measured by qPCR (n = 4). (E) Relative fecal *Akkermansia* DNA levels normalized to *eubacteria* levels with or without BFT under antibiotic treatment (abx+) and withdrawal of antibiotics for 3 weeks (abx −) (n = 8). *p < 0.05, **p < 0.01 by unpaired, 2-tailed t test.

We evaluated the effects of BFT on the intestinal motility in chow-fed mice. Migration of a charcoal meal in the intestinal tract was more rapid by 30% in the BFT group as compared to the control group (Fig. 2A), suggesting that BFT enhances gut motility. Since we observed an increase of the cecum size in the BFT group (Fig. 1H), the cecum size has been reported to be mediated by the gut microbiota^35,36^, we hypothesized that BFT modified the bacterial composition. Indeed, principal component analysis (PCA) of fecal samples, as assessed by 16S rRNA sequencing, revealed a clear separation in the microbial community structures between HFD and HFD + BFT mice. (Fig. 2B). BFT reduced the relative abundance of Bacteroidetes from 52% to 34%, whereas it markedly increased the amounts of Verrucomicrobia from 3.4% to 24% (Fig. 2C). Further analysis at the genus level revealed that the increase in Verrucomicrobia by BFT was mainly associated with a bloom of AKK (Fig. 2C and Supplementary Fig. 2). Time-dependent evaluation of AKK DNA in the feces revealed that a dramatic decrease of the AKK levels after a week of administration of an HFD, with restoration of the AKK levels following BFT administration for a week (Fig. 2D). When the endogenous intestinal bacteria were reduced by administration of an antibiotic cocktail for 2 weeks, the AKK bloom after 3 weeks of antibiotic discontinuation was more remarkable and 100,000 times higher in HFD + BFT group as compared to the level of HFD group (Fig. 2E). These results indicate that BFT modified the gut microbiota and in particular, caused a bloom of AKK.

### Administration of any single crude ingredient was insufficient to Elicit AKK bloom or improve the glucose metabolism

To further identify if any of the crude ingredients of BFT was mainly responsible for the microbiota modification by BFT, 11 major components of BFT were administered separately to mice, and the AKK DNA contents in the feces were compared. Whereas a week of an HFD with saline decreased the AKK level as compared to the pre-treatment level, several components such as Platycodi radix and Cnidii rhizoma seemed to increase the AKK level to some extent, but the degree was relatively small as compared to that observed in the mice administered BFT itself (Supplementary Fig. 3A). Furthermore, single administration of Platycodi radix or Cnidii rhizoma for 3 weeks did not induce an AKK bloom (Supplementary Fig. 3B). Furthermore, there was no significant difference in the glucose tolerance in the mice administered any component of BFT as compared to the HFD mice (Supplementary Fig. 3C). These results suggest that no single crude ingredient was directly responsible for the increase in the AKK levels or improvement of the glucose tolerance in the HFD mice treated with BFT, indicating that the combination of components of BFT contributed to the improvement of glucose metabolism as compared to any single component.

### BFT strengthened the gut barrier function and alleviated the endotoxemia in the HFD-fed mice

**Figure 3:**
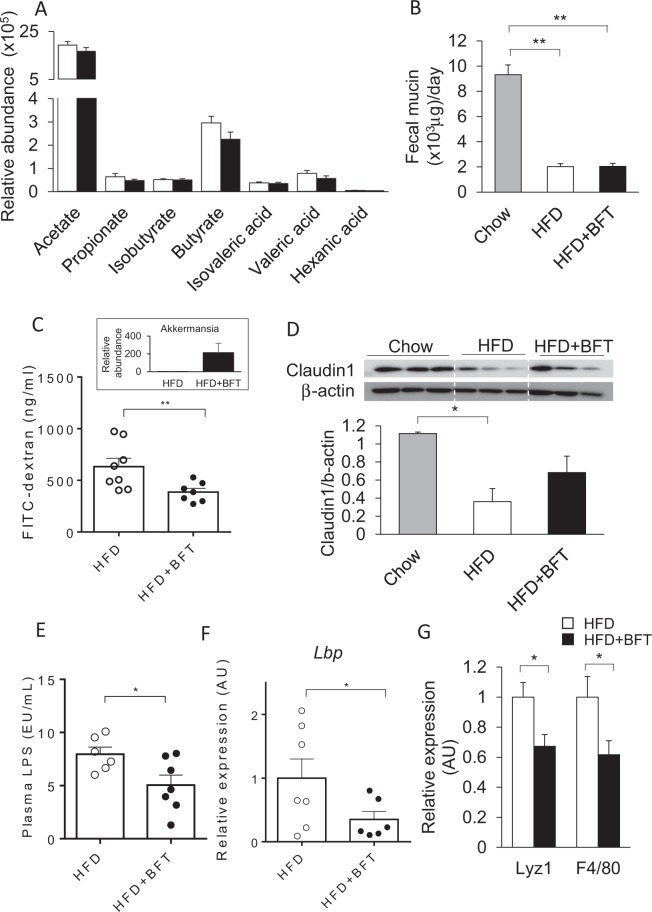
BFT increases tight-junction related protein in the colon and prevents from metabolic endotoxemia in HF-fed mice. (**A**) Relative short-chain fatty acid levels in the cecum of HFD (white) or HFD + BFT (black). (**B**) Fecal mucin content in mice treated either chow, HFD or HFD + BFT. (**C**) FITC levels in serum of mice after gavage administration of FITC-dextran. The Figure in the upper square is relative *Akkermansia* DNA levels of mice on a HFD or HFD + BFT used for the experiments (n = 7–8). (**D**) Western blots for claudin1 in the colon of mice on a chow or an HFD with or without BFT (18 weeks of age, 12 weeks on the HFD, 8 weeks on BFT) and quantitation of claudin1 protein normalized to β-actin (n = 3 per group). (**E**) Lipopolysaccharide (LPS) levels in serum of mice fed on an HFD with either saline or BFT for 12 weeks (n = 6–7). (**F**) *Lbp* mRNA levels in the liver (n = 7). (**G**) Lyz1 and F4/80 mRNA levels in the colon (n = 8–13). *p < 0.05 and **p < 0.01, by unpaired, 2-tailed t test for (**A,C,E,F,G**) or ANOVA, followed by Turkey-Kramer post-hoc for (**B**,**D**).

Several reports have shown that bacterial metabolites such as short chain fatty acids (SCFAs), have a considerable impact on the glucose metabolism. We checked the levels of SCFA in the cecal contents. None of the major components of SCFAs, such as acetate, propionate, isobutyrate, butyrate, isovaleric acid, valeric acid or hexanic acid, were altered in the BFT-treated mice, despite the alterations of the gut microbiota (Fig. 3A). Thus, the observed metabolic changes in the mice administered BFT were not associated with changes in the levels of SCFAs, which are the major bacterial metabolites. To further clarify the mechanisms underlying the improved insulin sensitivity induced by BFT, we examined the fecal mucin levels. Dramatic decrease of the mucin content was observed in the HFD mice, which was not affected by BFT treatment (Fig. 3B). Next, we examined the gut barrier function. Mice showing AKK bloom following BFT treatment showed lower serum levels of fluorescein isothiocyanate (FITC) after FITC-dextran gavage, suggesting decreased the gut permeability by BFT administration (Fig. 3C). While expression of the tight-junction-related protein, claudin-1, in the colon was significantly reduced in the HFD mice, BFT canceled the significance (Fig. 3D). Next, we investigated the effect of BFT on the endotoxemia in the HFD mice. The plasma endotoxin level as well as expression of the lipopolysaccharide binding protein (*Lbp*) in the liver were significantly decreased in the BFT-treated mice (Fig. 3E,F). To further confirm the effect of decrease in the gut permeability on the colonic microenvironment, we performed qPCR to assess the antimicrobial peptide and macrophage infiltration levels. In association with the enhanced gut barrier function, the expression of *Lyz1* and *F4/80* in the colon were significantly decreased in the HFD + BFT group as compared to the HFD + saline group (Fig. 3G). Taken together, BFT increased the tight-junction-related proteins, decreased the gut permeability and alleviated the endotoxemia in the HFD mice.

### BFT relieved adipose tissue inflammation and steatohepatitis

**Figure 4:**
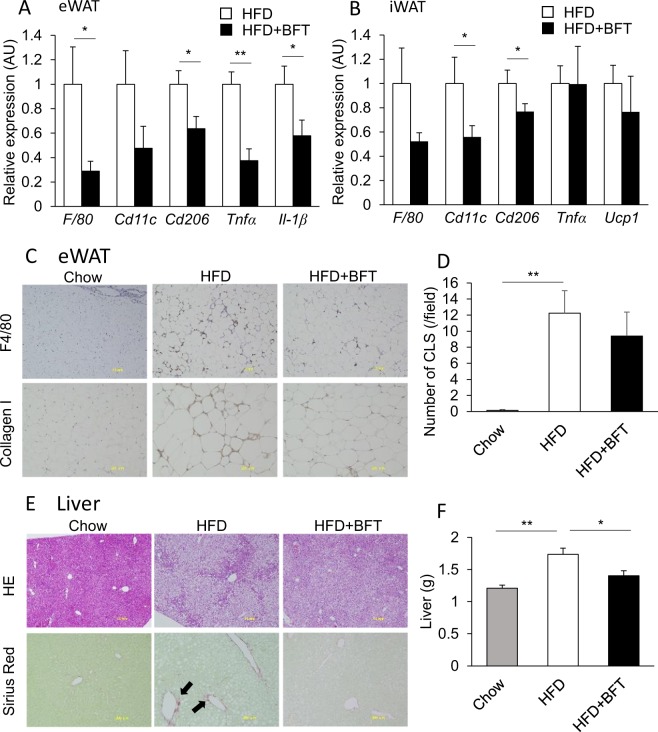
BFT decreases adipose tissue inflammation and improved steatohepatitis in HFD-fed mice. qPCR of macrophage and inflammation related genes in epididymal adipose tissues (**A**) and inguinal adipose tissue (**B**) of mice treated with either saline (white) or BFT (black) on an HFD for 8 weeks (n = 11–22). (**C**) Immunohistochemistry of epididymal adipose tissue for F4/80 and collagen I. (**D**) Quantification of crown like structures (CLS). (**E**) H&E staining and Sirius Red staining of the liver of chow, HFD or HFD + BFT treated mice. The arrows indicate Sirius Red positive area. (**F**) Liver weight of chow, HFD or HFD + BFT fed mice. *p < 0.05 and **p < 0.01, by unpaired, 2-tailed t test for (**A**,**B**), or ANOVA, followed by Turkey-Kramer post-hoc for (**D,F**).

Endotoxemia has been reported to promote the development of chronic inflammation of adipose tissue and steatohepatitis, leading to the development of insulin resistance. qPCR analysis revealed that BFT decreased macrophage and inflammatory gene expressions in both epididymal and inguinal adipose tissues (Fig. 4A,B). Immunohistochemical staining of epididymal white adipose tissue (eWAT) revealed apparent decrease in the accumulation of collagen I and abrogation of the increase in the number of crown-like structures (CLS), constituted mainly by inflammatory M1 macrophages, in the BFT-treated mice (Fig. 4C,D). In the liver, BFT administration decreased lipid droplets and fibrosis (Fig. 4E). Furthermore, BFT attenuated the increase in the liver size in HFD mice (Fig. 4F). Thus, BFT improved adipose tissue inflammation and steatohepatitis, with consequent alleviation of the endotoxemia induced by an HFD, and improvement in the glucose metabolism.

### Bacterial transplantation after BFT administration improved glucose metabolism

**Figure 5:**
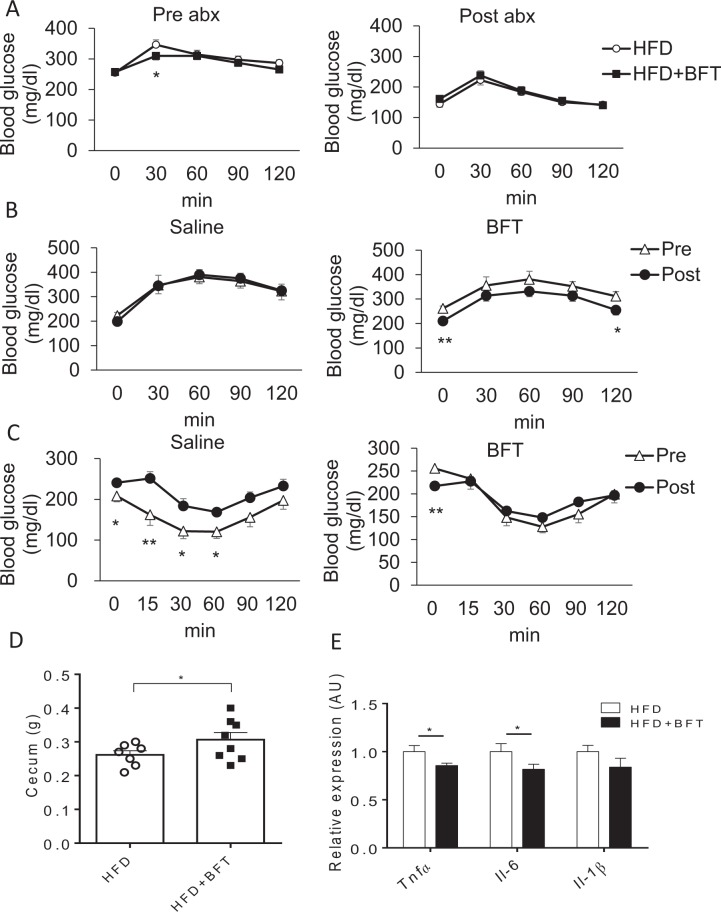
Improvement of glucose metabolism with decreased adipose tissue inflammation by BFT– modified gut microbiota is transferable. (**A**) OGTT of HFD-fed mice treated with or without BFT before (left) (15 weeks old) and after (right) antibiotic treatment for 3 weeks. (**B**) OGTT of the HFD-fed recipient mice performed before (triangle) (17 weeks old) and after (circle) (26 weeks old) bacterial transfer from mice treated with saline (left) or BFT for 2 weeks (right) (n = 8). (**C**) ITT of the HFD-fed recipient mice performed before (triangle) and after (circle) bacterial transfer from mice treated with saline (left) or BFT (right) (n = 8). (**D**) Cecum weight of recipient mice. (**E**) qPCR for inflammatory gene expressions in the epididymal adipose tissue of recipient mice (n = 8). *p < 0.05 and **p < 0.01, by unpaired, 2-tailed t test.

To examine the effect of gut microbiota modification by BFT on the glucose metabolism, antibiotics were administered to HFD or HFD + BFT mice. The OGTT revealed that the glucose tolerance improved by BFT was abrogated by the antibiotic treatment (Fig. 5A). To determine whether the improved metabolic phenotype observed in the BFT-treated mice was secondary to changes in the gut microbiota, we performed cecal microbiota transfer from HFD + saline and HFD + BFT donor mice into microbiota-depleted recipient mice. While the gut microbiota transplantation from the saline-treated mice had no effect on the glucose tolerance in the recipient mice, bacterial transfer from the BFT-treated mice improved the recipient mice’s glucose levels (Fig. 5B). Throughout the ITT period, the elevated blood glucose levels in the recipient mice improved to levels similar to those in the control group after intestinal bacterial transplantation from BFT-treated mice (Fig. 5C). This improved glucose metabolism in the recipient mice was associated with increase of the cecum size (Fig. 5D) and decreased inflammatory gene expressions, such as *Tnf-α* and *Il-6*, in the epididymal adipose tissue (Fig. 5E). Moreover, we performed bedding transfer three times weekly from the cages of the BFT-administered mice or control mice to those of the HFD + saline mice. Bedding transfer of the BFT-treated mice resulted in improved glucose metabolism, as determined by both OGTT and ITT, in the recipient cage mice (Supplementary Fig. 4A,B). These metabolic effects were associated with increased AKK levels in the feces of the recipient mice of the bedding of the BFT-treated mice (Supplementary Fig. 4C). These results indicated that the improvement in glucose metabolism by BFT was associated with modification of the gut microbiota by BFT, and that the effects could be reproduced by bacterial transplantation from the BFT-treated mice.

## Discussion

Bofutsushosan (BFT), is an oriental herbal medicine that has traditionally been used on patients with obesity and constipation in Asian countries. In this study, we clarified the beneficial effects of BFT on glucose metabolism. BFT modified the intestinal microbiota and enhanced the intestinal barrier function to alleviate obesity-related endotoxemia, consequently improving chronic inflammation and glucose tolerance. The favorable effects of BFT on metabolism were reproduced by transplanting the intestinal bacteria of mice receiving BFT and abrogated by antibiotic administration. These results indicate that changes in the intestinal bacterial composition induced by BFT contributed to the improvement of the glucose metabolism.

BFT has been clinically used since the 12th century. Several reports have shown that BFT administration decreases the body weight and improves glucose metabolism in both humans and mice^31–33^. BFT contains ephedrine, which enhances noradrenaline release, and licorice. At high doses, BFT activates *UCP-1* expression in the brown adipose tissue and decreases plasma ghrelin levels, suppresses food intake, suppresses body weight gain by decreasing the visceral adipose tissue, and attenuates the progression of steatohepatitis^30–34,37^. However, in our study, we found no effect of BFT on the body weight. Moreover, although the weight of BAT was decreased, there were no changes in the expression levels of *Pgc-1α* and *Ucp1*. These differences from previous reports could be attributable to differences in the protocols of BFT administration among studies.

We found that long-term BFT administration enlarged the cecum size. In mice, the composition of the gut microbiota can affect the cecum size due to the different bacterial fermentation rates. For instance, cecum sizes are smaller in HFD mice as compared to chow-fed mice, while germ-free mice and mice with decreased bacterial biomass by antibiotics have larger cecum sizes. These changes in the size of the cecum can be reproduced by bacterial transfer^36^. Indeed, 16S rRNA sequence analysis of fecal samples showed dynamic changes in the gut microbiota induced by BFT, including a bloom of *Akkermansia muciniphila* (AKK), a modifier of glucose metabolism. Initially, we hypothesized that the overall bacterial changes may underlie the improved glucose metabolism. We checked the cecal levels of SCFA levels, major microbial metabolites that can affect glucose metabolism. However, the microbiota modification by BFT was not associated with altered SCFA levels. Therefore, we focused on the effects of AKK bloom. It has been reported that high AKK levels are associated with improved obesity and related disorders, such as chronic inflammation, glucose intolerance, insulin resistance and steatohepatitis. AKK levels are decreased in obese subjects, and some intervention to increase AKK in these subjects was known to enhance the gut barrier function by thickening the mucous layer and increasing tight-junction-related protein in the intestinal epithelia, contributing to improved insulin sensitivity^23,26,28,38^. Plovier *et al*. recently reported that administration of a purified membrane protein from AKK can improve the gut barrier function and glucose metabolism^24^.

BFT alters the intestinal microbiota, and restores the tight-junction-related protein claudin-1 protein in the colon, which was associated with a bloom of AKK. As a result, the gut permeability was decreased and the endotoxemia caused by obesity was alleviated, with consequent improvement of the insulin resistance and glucose metabolism. Cecal microbiota transplantation from BFT-treated mice reproduced the improvement of the glucose metabolism. In general, changes in the gut microbiota by diet are often seen in about one week. The authors also confirmed that the differences in the donor cecum size could be reproduced in the recipient after two weeks of transplantation^36^. Based on these experiences, oral glucose tolerance tests were performed 3 weeks after the transplantation in this study. There is still a possibility that changes in the amount of other bacteria than just AKK may contribute to the improved glucose metabolism by BFT. However, our data suggested that the improved barrier function and decrease in systemic inflammation observed following BFT administration may be associated with increased AKK levels in the gut.

In regard to the doses of BFT used in this study, 25 mg/day/mouse was about 10 times as high as compared to the dosed used in humans. In mouse models, BFT has often been administered at the dose of 1–5% of the daily food intake^30–32^. Azushima *et al*. reported that diets containing 4.7% BFT elicited weight loss and metabolically favorable effects in mouse models by improving the functions of adipose tissue^31^. Ono *et al*. administered BFT and observed decreased weight gain at a 5% dose, but not at a 2% dose, whereas it attenuated the progression of NASH at both doses^30^. In a mouse study conducted by Akagiri *et al*., administration of 1% BFT to HFD mice had no effect on the degree of body weight gain, but decreased the adipocyte size in the adipose tissue^32^. Kobayashi *et al*. treated HFD-fed mice with BFT at 1.0, 1.5 and 2.0 g/kg/day for 25 days and observed anti-obesity effect at dose dependent manner^39^. Taken together, in mouse models, BFT exerts an anti-obesity effect only the dose of 5%, although it exerts other favorable effects on metabolism even at lower doses, suggesting that BFT has other effects than just an anti -obesity effect. We treated mice with 25 mg/day/mouse in this study, corresponding to 0.5–1% of the daily food intake and a rather lower dose as compared to that used in previous studies; even at this dose, however, we found that BFT improved glucose metabolism through modification of the gut microbiota.

In general, an HFD and obesity reduce the thickness of the mucous layer and deteriorate the intestinal barrier function. With this leaky -gut, lipopolysaccharides derived from gram-negative bacteria flow into the circulation. This so-called metabolic endotoxemia can promote insulin resistance^19^. Although BFT has neither been shown to alter the fecal mucin level nor to alter the amount of bacterial metabolites, such as short-chain fatty acids, it increased tight-junction-related protein claudin 1 level in the colon, leading to improved gut permeability and attenuated endotoxemia. In regard to the result that there was no change in the fecal mucin levels, one possible reason may be the increased intestinal motility induced by BFT, which is one of the clinical effects of BFT for constipation. This may cause increased mucin clearance and turnover in the intestine to diminish the difference in the mucin levels between the HFD and the HFD + BFT group^40^. However, the expression levels of the tight-junction-related protein, claudin-1, was restored by BFT administration. As a result, the gut barrier function improved with attenuation of metabolic endotoxemia. To further confirm the effect of decrease in the gut permeability on the colonic microenvironment, we performed additional qPCR to assess the antimicrobial peptide and macrophage infiltration levels. In response to improved gut barrier function by BFT, the expression levels of Lyz1, a marker of antimicrobial peptides, and F4/80 in the colon were significantly decreased following BFT administration. Further studies are needed to clarify the mechanisms underlying the enhanced expression of claudin-1 in the colon by BFT.

There are three possible mechanisms by which BFT modifies the gut microbiota. First, some components of BFT can directly stimulate the growth of certain bacterial species. Several herbal medicines which are found in BFT are known to be metabolized by intestinal bacteria. For example, glycyrrhizinate and baicalin, which are components of BFT, are known to be metabolized by intestinal bacterial enzymes, such as β-glucosidase and β-glucuronidase. Thus, intestinal bacteria are involved in the metabolism of active ingredients in herbal medicines, which could affect bacterial activities, and contribute to changes in the intestinal bacterial composition. Second, changes in the colonic microenvironment by BFT, such as the decrease in antimicrobial peptides, could be involved in the modification of the gut microbiota. AKK bloom may occur as a result of interactions with other bacteria. With regard to the importance of certain herbal ingredients in BFT, at least 11 major ingredients were found to exert only a minimal or no effect by themselves on the AKK levels, suggesting that it is a mixture of ingredients that may have some impact. As shown in Supplementary Fig. 5, BFT contains various chemical compounds, and further studies are essential to identify further compounds that could contribute to the increase in AKK levels. Third, increased intestinal peristalsis by BFT may alter the intestinal environment to provide a favorable environment for the growth of AKK. It is known that Rheum, which is one of the component herbs of BFT, promotes intestinal motility. In fact, we found that intestinal transport of charcoal meal was promoted by BFT. The enhanced intestinal movement may influence the intestinal environment and contribute to modification of the components of the microbiota. Furthermore, there could also be interactions among the three aforementioned mechanisms.

In recent years, many other medicines in the market, besides antibiotics, have been reported to have an impact on the gut microbiota^41^. Some of them affect glucose metabolism. For instance, metformin not only suppresses hepatic gluconeogenesis and enhances muscle glucose uptake, but also increases AKK and thickens the mucous layer, increasing the expressions of tight-junction-related proteins, thereby contributing to improved intestinal barrier function and glucose metabolism^28^. Similarly, some other medicines that exert pleiotropic and are used clinically to treat a variety of diseases can have an impact on the gut microbiota. Just as there are individual differences in the anti-obesity effects of BFT, there are generally individual differences in the effects of many drugs, which may be due to differences in the bacterial composition.

Our results showed that BFT alters the intestinal microbiota, strengthens the tight junctions in the intestinal epithelium, and alleviates endotoxemia caused by obesity, thereby improving glucose metabolism. Understanding the interaction between a medicine and the gut microbiota may lead to the finding of novel mechanisms of medical actions which can modify metabolism and, ultimately, improve health.

## Methods

### Mouse procedures

Bofutsushosan (BFT) (Lot Number: 2130062010) was kindly provided by TSUMURA & CO. (Tokyo, Japan). Male C57BL/6J mice were purchased from CLEA Japan at 5 week of age (Tokyo, Japan) and housed, four per cage, in an air-conditioned room with a controlled light/dark cycle (12 h/12 h) and food and water were available *ad libitum*. Since the microbial community structure is affected by the breeding conditions and BFT administration conditions, all mice are purchased from same vendor at same age and we acclimated the mice at our animal facility for a week before starting experiments. The mice were fed either a normal chow (Nosan Corporation, Yokohama, Japan) containing 10% of calories from fat, 26% from protein, and 64% from carbohydrates or an HFD (D12492, Research Diet) containing 60% of calories from fat, 20% from protein, and 20% from carbohydrates. After four weeks of HFD (10week of age), which induces body weight gain significantly, DIO mice were divided into two groups with BFT or control saline for 8 weeks. BFT was suspended in sterilized saline and orally administered with gavage daily (25 mg/mouse). For antibiotic treatment, mice were treated with a mixture of ampicillin (1 g/L) (Sigma-Aldrich), vancomycin (0.5 g/L) (LKT LAB), neomycin (1 g/L) and metronidazole (1 g/L) (Sigma-Aldrich) via the drinking water. For bacterial transfer experiments, donor mice were fed an HFD for a week and divided into two groups with BFT or control saline for 2 weeks. Cecal contents were collected from donor mice immediately after euthanasia, suspended with PBS, and filtered through a 40-μm cell strainer. Recipient mice were fed an HFD for 17 weeks and pretreated with the mixture of antibiotics for 3 days prior to the transfer. Bacterial transfer was performed by gastric gavage with 200 μl of diluted cecal contents. Oral glucose tolerance tests were performed before and 3 weeks after the bacterial transfer.

### 16SrRNA sequencing analysis

DNA was extracted from mouse feces using a MO BIO Fecal DNA Extraction Kit (QIAGEN). A multiplexed amplicon library converting the 16S rDNA gene V4 region was generated from DNA-extracted samples and the sequencing was performed in Repertoire Genesis Inc (Osaka, Japan). Principal component analysis (PCA) was performed using the prcomp command of the R version 3.2.1.

### Insulin and glucose tolerance test

Oral glucose tolerance test (2 g / kg weight) and intraperitoneal insulin tolerance test (1.0 unit / kg weight) were performed after 4-hour and 2-hour fasting period, respectively. Blood was collected from the tail vein at specific time points and glucose levels were measured using a STAT STRIP Express 900 (Nova Biomedical, Waltham MA).

### Evaluation of intestine motility

Animals were fasted for 4 h prior the experiment, but consumed water ad libitum. Mice were received the charcoal meal (10% charcoal in 5% gum 20 rabic). After 20 min, the mice were scarified and the intestinal tract was excised. The distance traveled by the charcoal meal from the pylorus was measured and expressed as a percentage of the total length of the small intestine from the gastro-pyloric junction to the ileocecal junction.

### Fecal mucin measurement

Fecal mucin level was determined on the basis of fluorometric measurement of *O*-linked reducing sugars^42^ using a commercially available kit (Cosmo Bio, Tokyo, Japan).

### Histological analysis

For the immunostaining of F4/80 and Collagen type I, eWATs fixed with 4% PFA were subjected as a paraffin embedded sample according to the standard procedure. Paraffin sections were deparaffinized and rehydrated, then were immunostained by rat monoclonal anti-F4/80 (1:500; Bio-Rad Laboratories, Hercules, CA) and rabbit polyclonal anti-collagen type I (1:250; Abcam), and were visualized using the appropriate Histofine Simple Stain Mice System (NICHIREI BIOSCIENCES, Tokyo, Japan) and 3,3′-diaminobenzidine tetrahydrochloride (DAB, Dako, Glostrup, Denmark) reaction.

For the histochemistry, livers fixed with 4% PFA were subjected as a paraffin embedded sample. Paraffin sections were deparaffinized and rehydrated, then were stained with Hematoxylin & Eosin or Sirius Red. Images were obtained by a microscopy system (BX 51; Olympus) connected to a digital camera (DP70; Olympus).

### Quantitative real-time PCR

Total RNA was isolated with an RNeasy mini kit (QIAGN), and complementary DNA (cDNA) was synthesized with TaKaRa PrimeScript RNA Kit (Takara, Shiga, Japan), according to the manufacturer’s protocol. The PCR amplification reaction was performed using gene-specific primers and TB Green Fast qPCR Mix (Takara, Shiga Japan). Fluorescence was monitored and analyzed in the Mx3000P QPCR System (Agilent Technologies, SantaClara, CA, USA) with an initial denaturation at 95 °C for 3 min, followed by 45 cycles of 10 s at 95 °C and 45 s at 60 °C. Each sample was run in duplicate and the relative mRNA levels were normalized to *Tf2b* mRNA. For relative abundances of AKK, total amount of bacterial DNA was normalized to eubacteria DNA. The data were analyzed according to the 2^ΔΔCT^ method. Primer sequences are listed in Supplementary Table 2.

### Plasma analysis

Plasma for endotoxin measurement was obtained by cardiac puncture and measured by Limulus Amebocyte Lysate Chromogenic Endopoint Assay (Hycult Biotech, Uden, NL) according to the manufacturer’s protocol. Insulin level was measured by Insulin ELISA Kit (Shibayagi, Japan).

### Western blotting

Proteins were extracted with 1x RIPA buffer containing 0.1% SDS. 18 μg of protein was subjected to SDS-PAGE and transferred to polyvinylidene fluoride membranes. Primary antibodies for β-actin (1:3000) were purchased from Cell Signaling Technology (Danvers, MA) and for claudin-1 was from Invitrogen (1:200). The secondary antibody conjugated to horseradish peroxidase (HRP) was purchased from GE Healthcare (Japan) (1:1000). The intensities of the bands were detected by using ChemiDoc Touch MP system (Bio-Rad). ImageJ software was used for a quantification.

### FITC-dextran experiment

FITC-dextran experiments were performed as described previously^17^. FITC-dextran (Sigma-Aldrich. St. Louis, MO) was orally administered. Four hours after gavage, blood samples were collected by cardiac puncture. The plasma was diluted 1:1 (vol/vol) in PBS and fluorescence intensity of each sample was measured (excitation: 485 nm, emission: 528 nm) with fluorescence spectrometer. FITC-dextran concentrations were calculated from a standard curve.

### Measurement of cecal SCFAs

Extraction and measurement of SCFAs in cecal samples was described elsewhere^43^. Briefly, the extraction was performed using water, concentrated hydrochloric acid, and diethyl ethel, and the derivatization of samples was carried out using *N*-tert-butyldimethylsilyl-*N*-methyltrifluoroacetamide (MTBSTFA) followed by the heating at 80 °C for 20 min. Levels of SCFA were determined by using Agilent 5977 MSD single Quad mass spectrometer coupled to Agilent 7890 Gas Chromatography (GC/MS) with selected ion monitoring (SIM) mode. GC/MS conditions were described previously^44^. Quantification of SCFA levels was calculated by integrated sum of area using Mass Hunter Quantitative software (Agilent).

### Experimental animal ethics statement

All animal studies and experiments were performed in accordance with guidelines provided and approved by the Animal Experiment Ethics Committee of the University of Toyama (#A2016MED-31).

### Statistics

Statistical significance was evaluated using ANOVA, followed by a Turkey-Kramer post-hoc or unpaired, two-tailed *t* test where appropriate. A *P* value of less than 0.05 was considered significant. Results are presented as the mean ± SEM.

## Supplementary information

Supplementary Information.

## Data Availability

The datasets generated and analyzed during the current study are available from the corresponding author upon reasonable request.
