# Supplementary material for: Bofutsushosan improves gut barrier function with a bloom of Akkermansia muciniphila and improves glucose metabolism in mice with diet-induced obesity
Source: Sci Rep. 2020 Mar 26;10:5544. doi: 10.1038/s41598-020-62506-w (PMC7099031; doi:10.1038/s41598-020-62506-w)
Supplement: Supplementary file 1 — Supplementary Information. [file 41598_2020_62506_MOESM1_ESM.pdf]

Supplementary Information

Bofutsushosan improves gut barrier function with a bloom of *Akkermansia muciniphila* and improves glucose metabolism in mice with diet-induced obesity

Shiho Fujisaka[1), Isao Usui[1)2)], Allah Nawaz[1)3)], Yoshiko Igarashi[1)], Keisuke Okabe[1)4)], Yukihiro Furusawa[5)], Shiro Watanabe[6)], Seiji Yamamoto[7)], Masakiyo Sasahara[7)], Yoshiyuki Watanabe[1)], Yoshinori Nagai[8)], Kunimasa Yagi[1)], Takashi Nakagawa[3)], Kazuyuki Tobe[1)]

1) First Department of Internal Medicine, Faculty of Medicine, University of Toyama, Japan
2) Department of Endocrinology and Metabolism, Dokkyo Medical University, Japan
3) Department of Metabolism and Nutrition, Graduate School of Medicine and Pharmaceutical Sciences for Research, University of Toyama, Japan
4) Department of community Medical Support, Toyama University Hospital
5) Department of Liberal Arts and Sciences, Faculty of Engineering, Toyama Prefectural University, Japan
6) Division of Nutritional Biochemistry, Institute of Natural Medicine, University of Toyama, Japan
7) Department of Pathology, University of Toyama, Japan
8) Department of Pharmaceutical Engineering, Faculty of Engineering, Toyama Prefectural University, Japan

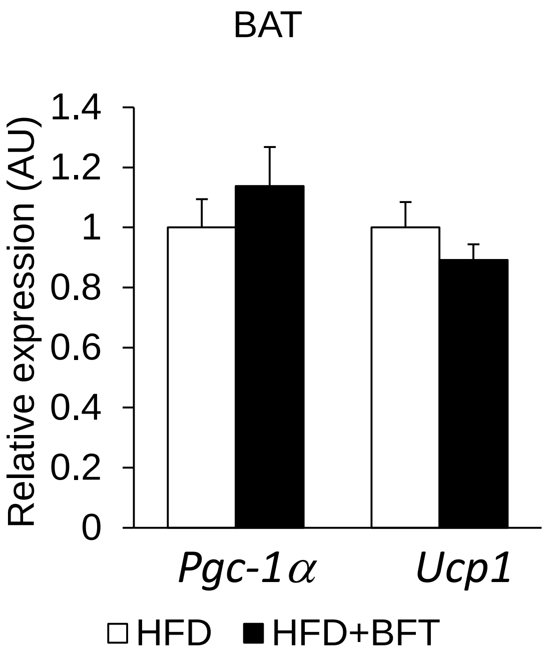

Supplementary Figure 1

Pgc1α and *Ucp1* expression in the BAT of mice on an HFD or HFD+BFT (18 week on age, n=9-11).

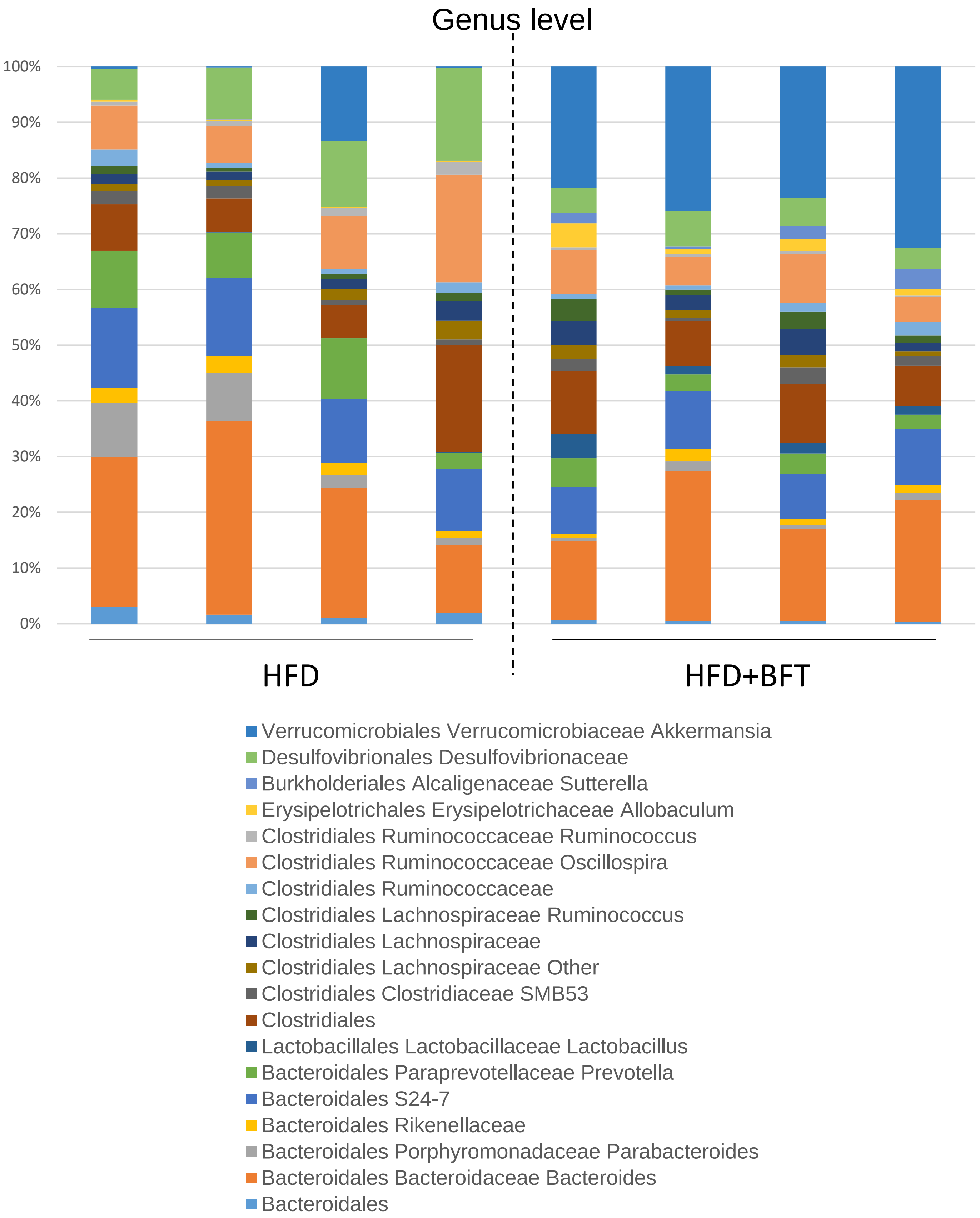

Supplementary Figure 2
Visualization of relative abundance of bacteria for each individual mouse at 19 weeks of age (n=4). Bacteria present more than 1% were shown.

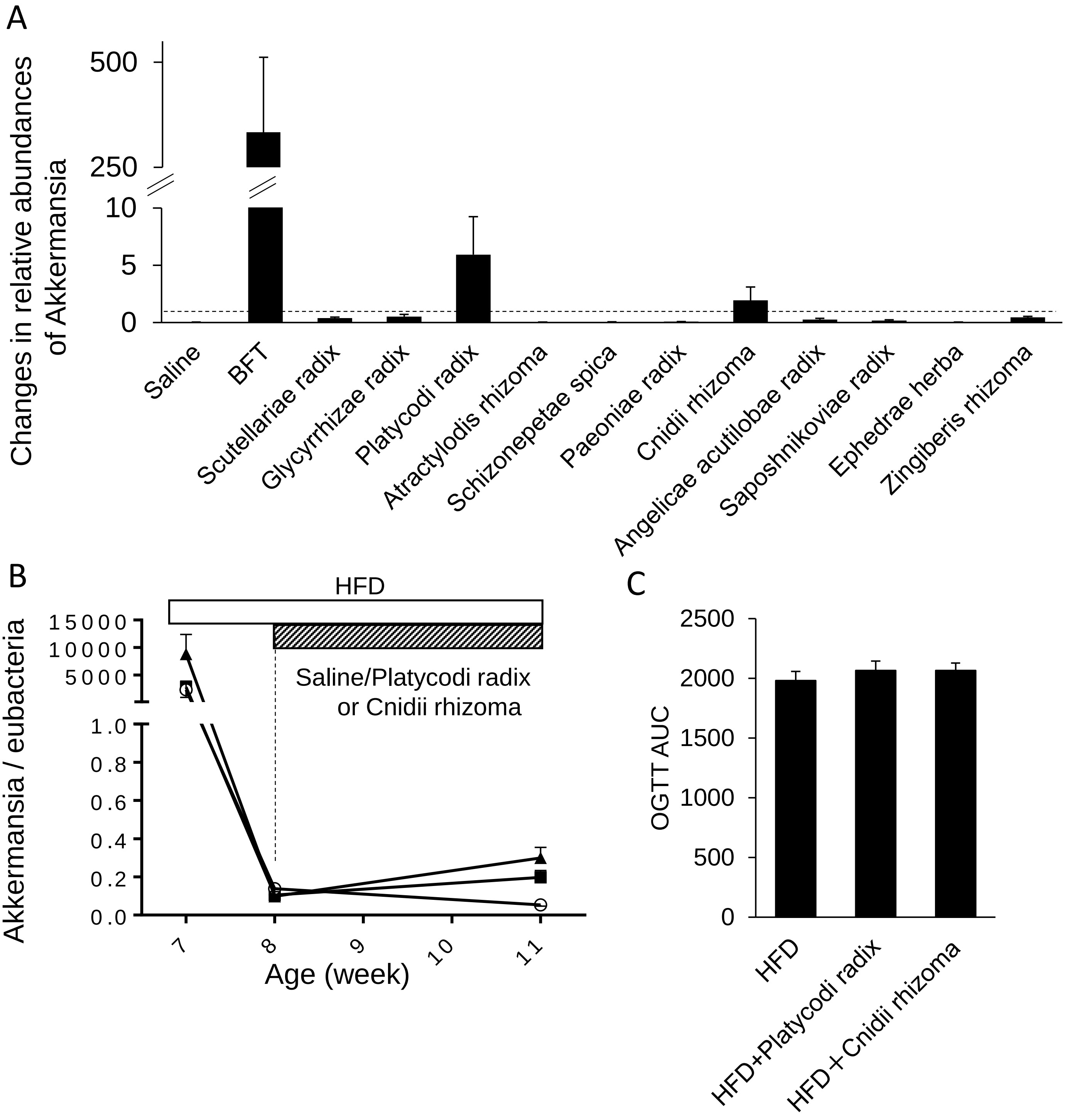

Supplementary Figure 3
(A)Changes in relative abundances of Akkernansia DNA in the feces. Mice were fed an HFD at 6 week of age.
Saline, BFT or 11 major ingredients were administered orally with gavage daily.
Feces from each mouse were collected before and a week after the administration. (n= 4)
(B) Relative abundances of AKK DNA in the feces. After a week of HF-feeding, mice were administered either
saline (circle), Platycodi radix (square), or Cnidii rhizoma (triangle). Feces of each mice were collected at 7,8 and 11 week of age.
(n=8)
(C) Area under curve during OGTT at 14 week of age (n=8).

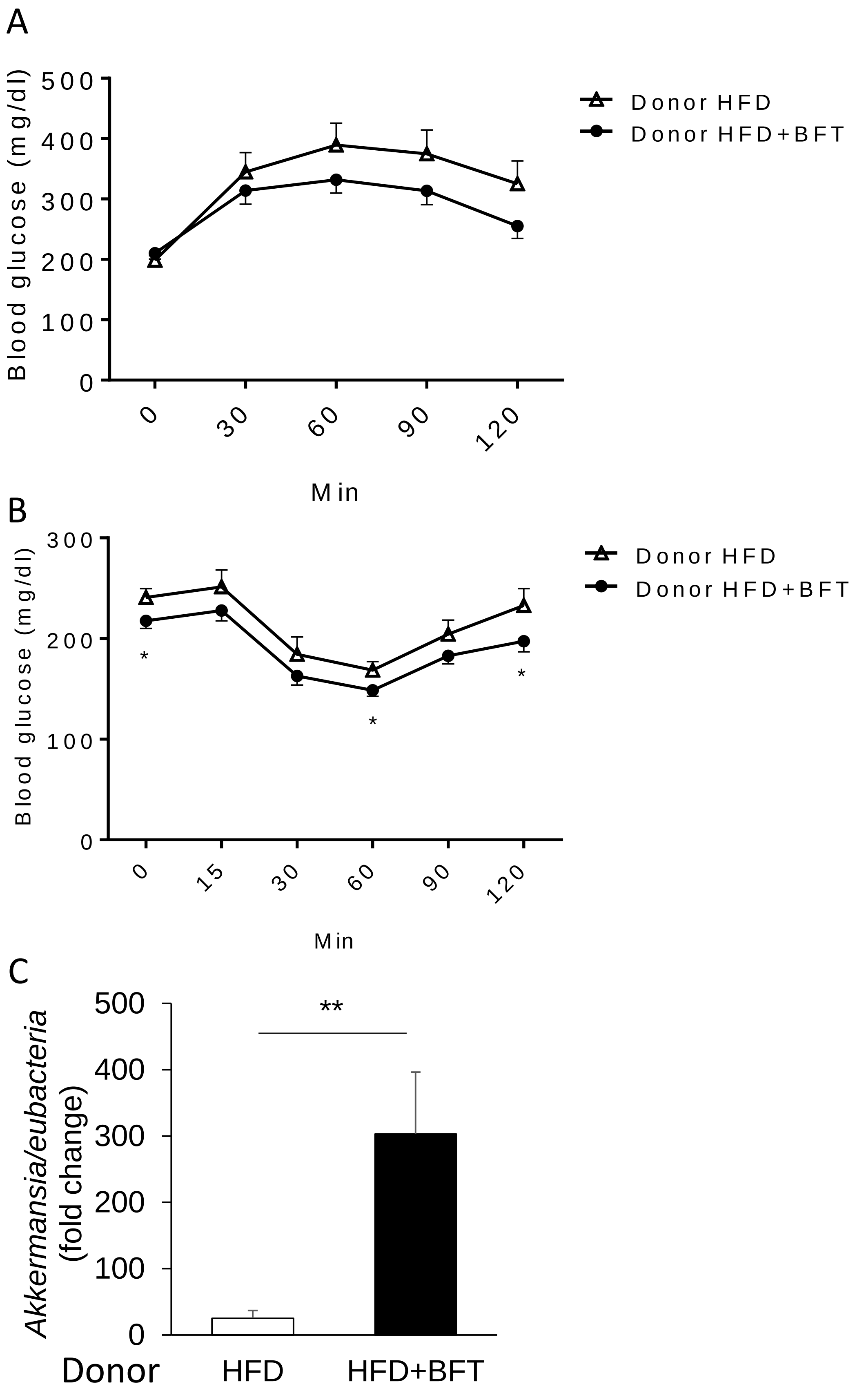

Supplementary Figure 4

OGTT (A) and ITT (B) of mice after bedding transfer (13 week old, n=8~9). Donor mice were treated with either an HFD +saline or HFD+BFT at 6 week of age. The recipient littermate mice were fed an HFD. Bedding of donor cages were transferred to the recipient cages 3 times a week and continued until 14 week old. (C) Relative AKK levels in the feces of recipient mice at 14 week old (8 weeks of bedding transfer) (n=8~9). *$p < 0.05$, by unpaired, 2-tailed t test.

HPLC pattern of BFT

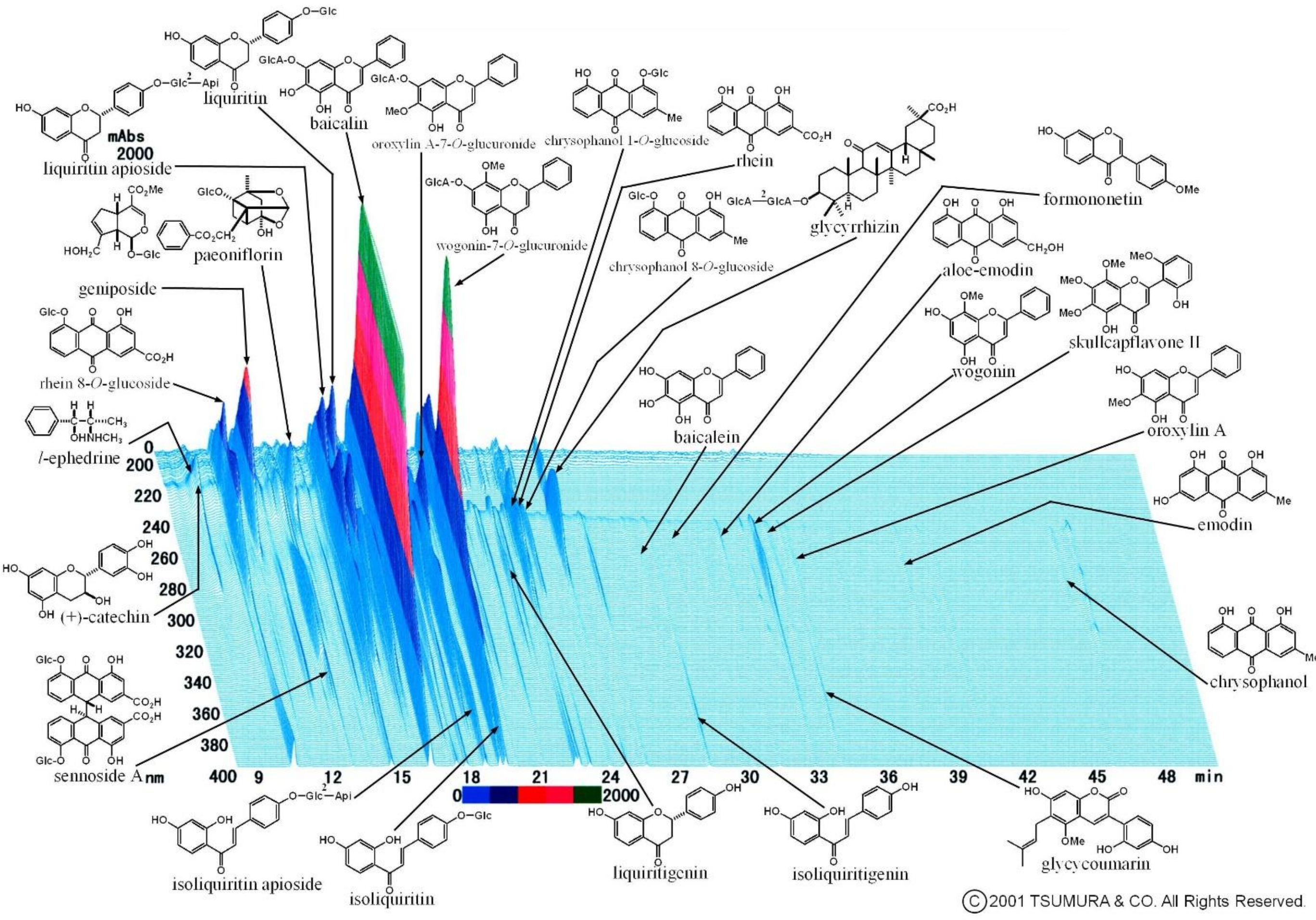

Supplementary Figure 5
Three-dimensional high-performance liquid chromatography of the major chemical compounds in BFT.

Crude components	Major ingredients	Weight ratio (g)
Kasseki	Aluminum silicate	3
Scutellariae radix	Flavonoid	2
Glycyrrhizae radix	Glycyrrhizic acid, Triterpenoid	2
Platycodi radix	Saponin, Inulin	2
Gypsum fibrosum	Calcium sulfate	2
Atractylodis rhizoma	Sesquiterpenoid	2
Rhei rhizoma	Anthraquinone	1.5
Schizonepetae spica	Monoterpenoid	1.2
Gardeniae fructus	Iridoid	1.2
Paeoniae radix	Monoterpenoid	1.2
Cnidii rhizoma	Phthalide	1.2
Angelicae acutilobae radix	Phthalide	1.2
Menthae herba	Monoterpenoid	1.2
Saposhnikoviae radix	Chromone	1.2
Ephedrae herba	Ephedrine	1.2
Forsythiae fructus	Triterpenoid	1.2
Zingiberis rhizoma	Pyrocatechol	0.3
Sal milabilis anhydricus	Sodium sulfate	0.7

Supplementary Table 1
18 crude components and major ingredients of BFT.

Gene	Forward	Reverse
Akkermansia	CAGCACGTGAAGGTGGGGAC	CCTTGCGGTTGGCTTCAGAT
Eubacteria	ACTCCTACGGGAGGCAGCAGT	ATTACCGCGGCTGCTGGC
Tf2b	TGGAGATTTGTCCACCATGA	GAATTGCCAAACTCATCAAAACT
F4/80	CTGGGATCCTACAGCTGCTC	AGGAGCCTGGTACATTGGTG
Cd11c	CTGGATAGCCTTTCTTCTGCTG	GCACACTGTGTCCGAACTCA
Cd206	TGATTACGAGCAGTGGAAGC	GTTCACCGTAAGCCCAATTT
Tnfα	ACGGCATGGATCTCAAAGAC	AGATAGCAAATCGGCTGACG
Il-6	TAGTCCTTCCTACCCCAATTTCC	TTGGTCCTTAGCCACTCCTTC
Il-1β	GCAACTGTTCCTGAACTCAACT	ATCTTTTGGGGTCCGTCAACT
Ucp-1	AGGCTTCCAGTACCATTAGGT	CTGAGTGAGGCAAAGCTGATTT
Pgc-1α	AACCACACCCACAGGATCAGA	TCTTCGCTTTATTGCTCCATGA
Lbp	GGCTCTGCAGAGAGAGCTGTACAA	TAGTTAAGGAATGCCTGGAACAGG

Supplementary Table 2

Figure 3D
Uncropped

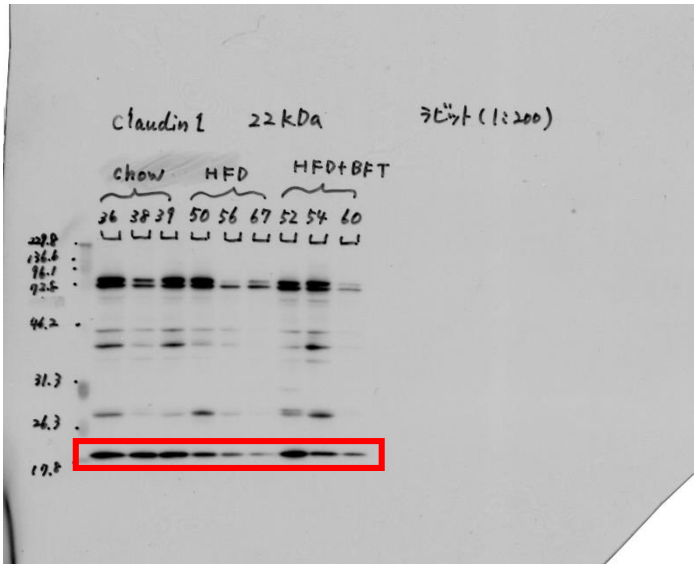

Claudin 1

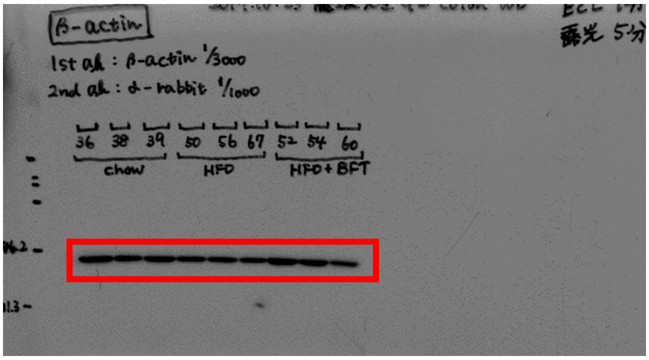

β-actin
